# Alpha-linolenic acid pretreatment alleviates NETs-induced alveolar macrophage pyroptosis by inhibiting pyrin inflammasome activation in a mouse model of sepsis-induced ALI/ARDS

**DOI:** 10.3389/fimmu.2023.1146612

**Published:** 2023-03-27

**Authors:** Chenchen Liu, Yu Zhou, Qing Tu, Liangfang Yao, Jinbao Li, Zhongwei Yang

**Affiliations:** ^1^ School of Anesthesiology, Weifang Medical University, Weifang, China; ^2^ Department of Anesthesiology, Shanghai General Hospital, Shanghai Jiaotong University School of Medicine, Shanghai, China

**Keywords:** sepsis, ARDS, NETs, pyrin, pyroptosis, alpha-linolenic acid (ALA)

## Abstract

**Background:**

Neutrophil extracellular traps (NETs) can cause acute lung injury (ALI)/acute respiratory distress syndrome (ARDS) by inducing macrophage pyroptosis. The purpose of this study was to find out whether pretreatment of alpha-linolenic acid (ALA) could inhibit NETs-induced macrophage pyroptosis in sepsis-induced ALI/ARDS, as well as to identify which inflammasome is involved in this process.

**Methods:**

LPS was instilled into the trachea to establish sepsis-induced ALI/ARDS in a mouse model. ​Lung injury was assessed by microscopic examination of lung tissue after hematoxylin and eosin staining, pathology score, and bronchoalveolar lavage fluid (BALF) total protein concentration. The level of NETs in lung tissue was detected by MPO-DNA ELISA. Purified NETs, extracted from peritoneal neutrophils, induced macrophage pyroptosis *in vitro*. Expression of pyroptosis-related proteins (Cl-caspase-1, Cl-GSDMD, ASC) and IL-1β in the lung tissue and bone marrow-derived macrophages (BMDMs) were determined by western blotting or ELISA. Specks of Pyrin/ASC were examined by confocal immunofluorescence microscopy. Mefv (Pyrin)^-/-^ mice were used to study the role of Pyrin in the process of sepsis-induced ALI/ARDS.

**Results:**

ALA alleviated LPS-induced lung injury. ALA reduced the level of NETs, pyroptosis-related proteins (Cl-caspase-1, Cl-GSDMD, ASC), and IL-1β in the lung tissue of sepsis mice. *In vitro*, NETs increased the expression of pyroptosis-related proteins (Cl-caspase-1, Cl-GSDMD, ASC) and IL-1β significantly in BMDMs. Pyrin protein was found to be higher and form the inflammasome with ASC in NETs challenged-BMDMs. Knockout of Mefv (Pyrin) gene fully restored the increased expression of pyroptosis-related proteins (Cl-caspase-1, Cl-GSDMD, ASC) and IL-1β *in vitro* and *in vivo*. Lung injury was alleviated significantly in Mefv (Pyrin)-/- mice as well.​ ALA suppresses all the NETs-induced changes as mentioned above.

**Conclusion:**

Our study is the first to demonstrate Pyrin inflammasome driving NETs-induced macrophage pyroptosis, and ALA may reduce ALI/ARDS by inhibiting the activation of the Pyrin inflammasome-driven macrophage pyroptosis.

## 1 Introduction

Sepsis-induced acute lung injury (ALI)/acute respiratory distress syndrome (ARDS) is a common disease with significant morbidity and mortality in ICU patients (1, 2). Despite the large number of studies on ALI/ARDS that have emerged in recent years, there is no specific treatment for ALI/ARDS, only supportive care such as pulmonary protection ventilation (3). Therefore, it is essential to explore the potential mechanism and treatment strategy of ALI/ARDS.

A diffuse inflammatory reaction in the lung tissue mainly characterizes sepsis-induced ALI/ARDS (4). Massive inflammatory cell infiltration, including neutrophils and macrophages, was previously known as a primary pathological feature of ALI/ARDS (5). In our previous research and others (6–8), macrophages were reported to undergo pyroptosis, releasing large amounts of pro-inflammatory factors, mainly IL-1β, thereby exacerbating the disease process of ALI/ARDS. The driving factors for macrophage pyroptosis and its underlying mechanism in ALI/ARDS remain unclear.

It is well known that neutrophils are the first line of the immune system against the invasion of pathogens and are recruited into the focus when tissue is inflamed (9). In this process, neutrophils receive stimulation and release reticular substances composed of chromatin and granular protein-neutrophil extracellular traps (NETs). NETs can capture and kill pathogens to some extent and play a particular anti-inflammatory role. Exaggerated and prolonged activation of neutrophils may contribute to ALI/ARDS despite their importance in targeting microbial agents (10, 11). Furthermore, some studies have found that excessive release of NETs can stimulate alveolar macrophages to undergo pyroptosis and mediate severe inflammatory cascade (12, 13).

Pyroptosis was driven by the inflammasomes. It has been shown that there are multiple families of pattern recognition receptors (PRRs) that form inflammasomes, such as nucleotide-binding domain and leucine-rich repeat receptors (NLRs), the absent in melanoma 2-like receptors (ALRs), and Pyrin (14). In response to specific pathogen-associated molecular patterns (PAMPs), certain PRRs recruit the adaptor protein apoptosis-associated speck like protein (ASC). Upon coupling with PRR, ASC can trigger caspase-1 to become active and cleave the pro-inflammatory cytokines, like pro-interleukin-1beta(IL-1β)and pro-interleukin-18(IL-18) (15). Further, inflammasome-associated caspases can cleave and activate gasdermin D (GSDMD) to form membrane pores for cytokine release and pyroptosis (16). Previous studies mainly focused on NLRs and ALRs. However, Pyrin, encoded by the Mediterranean fever gene (Mefv), also plays a role in mediating inflammasome (17). Whether pyrin functions as an inflammasome in NETs-induced macrophage pyroptosis is currently unknown.

Alpha-linolenic acid (ALA) is a plant-based omega-3 fatty acid (18). As an essential fatty acid for the human body, it has an incomparable effect on human health (19). It significantly prevents cardiovascular and cerebrovascular diseases, anti-lipid, anti-oxidation, anti-metabolic syndrome, and anti-cancer (20). ​The role of ALA in anti-inflammation has been increasingly demonstrated in recent years (21, 22). Our previous study also found that ALA can alleviate LPS-induced ALI by reducing the pyoroptosis-releated cytokines, IL-1β (23). However, whether ALA will further play an anti-inflammatory role by reducing pyroptosis remains unclear.

​Therefore, this study aims to explore whether ALA can suppress pyroptosis levels in lung macrophages, thus alleviating lung injury, and elucidate its underlying mechanism. Our study is the first to demonstrate Pyrin inflammasome driving NETs-induced macrophage pyroptosis, and ALA may reduce ALI/ARDS by inhibiting the activation of the Pyrin inflammasome-driven macrophage pyroptosis.

## 2 Materials and methods

### 2.1 Animals

Male C57BL/6 background mice of 6-8 weeks were purchased from Jihui Laboratory Animal Care Co., Ltd (Shanghai, China). Mefv (Pyrin) ^-/-^ mice were constructed by Cygen Biosciences (Suzhou, China) and bred by the Animal Laboratory of the First People’s Hospital affiliated with Shanghai Jiaotong University. All mice were raised in a pathogen-free environment with appropriate temperature and humidity, accepted circadian circulation, and freely obtained food and water.

All animal experiments were conducted according to the Guide for the Care and Use of Laboratory Animals and approved by the Ethics Committee of Shanghai General Hospital, Shanghai Jiaotong University School of Medicine (No. 2019AW009).

### 2.2 ALI/ARDS model establishment

Male C57BL/6 mice aged 6-8 weeks were randomly divided into PBS, ALA, LPS, and LPS+ALA groups. Under sevoflurane inhalation anesthesia, the midline skin of the mouse neck was cut off, the trachea was exposed until the trachea was visualized, and LPS (10mg/kg, diluted to 50ul with PBS; #L2880, Sigma-Aldrich) was instilled into the trachea with insulin needle to construct ALI/ARDS model. The mice in the PBS group were given the same amount of PBS liquid without LPS by intratracheal instillation in the same way. ALA (1800mg/kg; #L2376, Sigma-Aldrich) was injected intraperitoneally 1 hour (h) before surgery. All mice were anesthetized with 4% chloral hydrate to obtain bronchoalveolar lavage fluid (BALF) and lung tissue at 24 h after modeling.

### 2.3 Histopathological analysis

For pathological analysis of lung tissue, the left lung was fixed in 4% paraformaldehyde for 48 h, and then the lung tissue was dehydrated, and paraffin embedded. The paraffin tissue block was cut into 4 μm thick tissue sections, stained with hematoxylin and eosin (HE), and photographed under the optical microscope. The damage assessment was carried out by two researchers who did not know the experimental design. The assessment principle was based on a semi-quantitative scoring system.

### 2.4 Flow cytometry assay

Alveolar macrophages in BALF were purified with a MagniSort™ Mouse F4/80 Positive Selection Kit (Therma Fisher, 8802-6863-74) according to the manufacturer’s instructions. Then, cell suspensions were stained with Sytox (1:2000, Invitrogen, S7020) for 15 min at room temperature. All samples were acquired on a BD C6 flow cytometer (BD Bioscences) and analyzed with FlowJo software. The gating strategy of the cells is shown in **Supplementary Figure 3**.

### 2.5 Isolation and culture of bone marrow-derived macrophages

BMDMs were obtained as described in the previous study (24). The adult mice were humanely sacrificed and then soaked in 75% alcohol. The femurs and tibias were harvested from both sides, and the bone marrow was washed out with sterile precooled 1640 medium (Gibco). The red blood cells in medium were sufficiently separated by RBC lysis buffer. The finally harvested cells were cultured in 1640 complete medium containing 10% fetal bovine serum (FBS, Gibco), 50ng/ml penicillin/streptomycin, and 10 ng/ml recombinant macrophage-colony stimulating factor (M-CSF,315-02, PeproTech). The fresh medium was replaced on the 3^rd^ and 5^th^ day. The cells were used for subsequent experiments until they matured on the 7^th^ day.

### 2.6 PMNs isolation and culture

As described in previous studies (25), male mice aged eight weeks were intraperitoneally injected with 1ml casein solution (9 g casein, 0.5 mM Mgcl_2_ and 0.9 mM Cacl_2_ were dissolved in 100ml hot PBS) overnight. The peritoneal lavage solution was harvested 3 h after the second injection. The harvested precipitation was washed three times, and the discontinuous density gradient centrifugation was carried out with Percoll solution (P1644, Sigma). Purified peritoneal neutrophils were cultured in 1640 complete medium containing 10% fetal bovine serum and 50 ng/ml penicillin/streptomycin for the next step of NETs extraction.

### 2.7 NETs extraction

The purified peritoneal neutrophils were incubated with PMA (500nM, P1585, Sigma) for 4 h in the incubator (37°C, 5% CO_2_) (26). The culture medium was gently aspirated and discarded. Then the cell layer at the bottom of the culture dish was fully washed with 15 ml of precooled PBS without calcium and magnesium. The PBS suspension was centrifugated at 450xg, 10 min, 4°C. The supernatant liquid without cells was harvested and centrifugated at 18000xg, 10min, 4°C. The obtained precipitate is NETs particles. The particles were resuspended with 100ul PBS, quantified with a microspectrophotometer, and stored in a refrigerator at - 80°C for subsequent experiments.

### 2.8 Detection of IL-1β and protein concentration in BALF

The harvested BALF and cell supernatant were centrifuged to discard the sediment. The level of IL-1β was detected with an ELISA kit (EK201B, MULTI SCIENCES) according to the manufacturer’s instructions. Similarly, according to the manufacturer’s instructions, we determine the total protein concentration in BALF using a BCA Protein Assay Kit (P0012, Beyotime Biotechnology).

### 2.9 MPO-DNA complex enzyme-linked immunosorbent assay

As described in the previous study (27), 96 well flat plate was pre-coated with anti-MPO monoclonal antibody (1:500, SAB1409321, Merck Millipore) and incubated overnight at 4°C. The plate was then blocked with 1% BSA at room temperature. The samples with the incubation buffer containing peroxidase-labeled anti-DNA antibody(1:25, Cell Death ELISA PLUS, Roche)were added to the plate for 2 h at room temperature. ​The TMB substrate buffer was used to develop the color in darkness for 30 min. ​The absorption was detected at 450nm wavelength.

### 2.10 Western-blot analysis

BMDMs and lung tissue were lysed with RIPA lysis buffer (WB3100, NCM) and centrifuged (12000rpm, 10min, 4°C). The protein concentration was detected by the BCA protein detection kit according to instructions. Next, we used 10% SDS-PAGE to separate the protein lysates and transfer them to the PVDF membrane. The membrane was sealed with 5% skimmed milk for 2 h at room temperature, washed by TBST three times, and then we incubated the membrane with primary antibody overnight at 4°C. Primary antibodies include GSDMD (1:2000, #39754, CST), cleaved-GSDMD (1:2000, #10137, CST), caspase-1 (1:2000, #24232, CST), cleaved-caspase-1 (1:2000, #89332, CST), NLRP3 (1:2000, #15101, CST), ASC (1:2000, #67824, CST), Pyrin (1:2000, ab195975, Abcam). Similarly, after washing with TBST three times, the membrane and the secondary antibody were incubated at room temperature for 2h. The immunoreactive bands were exposed through the BIORAD ChemiDoc XRS system, and the Image J software was used for band analysis.

### 2.11 Immunofluorescence staining

BMDMs were inoculated into a 24-well plate covered with sterile cover slides. After cell differentiation and maturation, the cells were treated differently according to the experimental scheme. The treated cells were fixed by paraformaldehyde (4%), infiltrated by Triton X-100 (0.5%) and sealed by bovine serum albumin (BSA, 1%), and then incubated with primary antibody overnight at 4°C or Sytox (1:2000, Invitrogen, S7020) at room temperature for 15 min. The primary antibodies are listed as follows: ASC (1:200, ab175449, Abcam), Pyrin (1:200, ab195975, Abcam). After washing three times, the cells were incubated with the secondary antibody, if necessary, at room temperature for 2h in the dark. Next, the cells were co-incubated with DAPI for the patch. Finally, Leica SP8 confocal microscope was used to take pictures.

### 2.12 Statistical analysis

All data are represented by means ± standard deviation (S.D.) and analyzed by GraphPad Prism. Double-tailed Student’s t-test was used to compare data between two groups, and one-way ANOVA (with Tukey’s multiple comparisons test) was used to compare data between three groups or more. It is considered that when the P value is less than 0.05, the difference is statistically significant.

## 3 Result

### 3.1 ALA inhibited LPS-induced ALI/ARDS by reducing NETs production and macrophage pyroptosis in lung tissue

**Figure 1:**
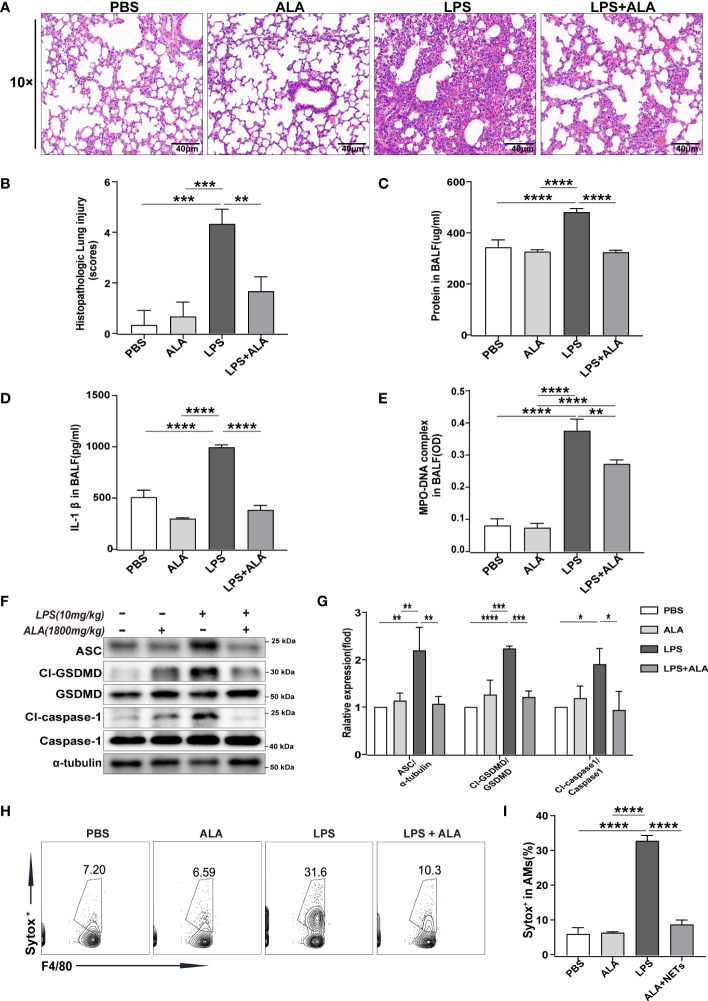
ALA inhibited LPS-induced ALI/ARDS by reducing NETs production and cell pyroptosis in lung tissue. Mice were subjected to ALI/ARDS by intratracheal instillation of LPS (10 mg/kg) and injected intraperitoneally with ALA (1800 mg/kg) or PBS 1 h before LPS instillation. At 24 h after LPS instillation, **(A)** haematoxylin and eosin (HE) staining lung tissue sections were examined histologically; **(B)** lung injury pathology score was assessed; **(C)** total protein concentration in BALF was determined by a BCA protein assay kit; **(D)** level of inflammatory cytokine IL-1β in BALF was detected by ELISA; **(E)** level of NETs in BALF was determined by MPO-DNA complex ELISA; **(F, G)** expression of pyroptosis-related proteins (Cl-caspase-1, Cl-GSDMD, ASC) in lung tissue was analyzed by western blotting; **(H, I)** death of macrophage in BALF was investigated by flow cytometry. Data were represented as mean ± S.D. (n = 3). *P < 0.05, **P < 0.01, ***P<0.001, ****P < 0.0001.

​To investigate the role of ALA in LPS-induced ALI/ARDS, we administered ALA intraperitoneally 1 h before intratracheal administration of LPS in mice (14). Using H&E staining of lung tissue, we found that LPS induced lung injury, manifested by severe interstitial edema and inflammatory cell infiltration. After ALA treatment, the degree of lung injury was significantly reduced (**Figure 1A**). The pathological score of lung injury also showed the same result (**Figure 1B**). In addition, the quantitative analysis of protein in BALF by BCA method showed that ALA could effectively reduce LPS-induced protein exudation in the lung tissue (**Figure 1C**). ALA was also found to inhibit the level of inflammatory cytokine IL-1β in BALF (**Figure 1D**). All the above results indicate that ALA may alleviate the LPS-induced lung injury.

To ​determine the underlying mechanism of ALA in relieving lung injury, we used MPO-DNA ELISA to analyze the level of NETs in BALF quantitatively. The results showed that with stimulation of LPS, the level of NETs in BALF increased significantly in both LPS and LPS + ALA groups compared to the PBS group. Compared to the LPS group, the LPS+ALA group has significantly lower levels of NETs (**Figure 1E**). Furthermore, we found that LPS enhanced the expression of pyroptosis-related proteins (Cl-caspase-1, Cl-GSDMD, ASC) in lung tissue significantly, which were eliminated by ALA treatment (**Figures 1F, G**). ​To investigate the role of ALA in alveolar macrophages, flow cytometry was used to detect the death of alveolar macrophages isolated from BALF. And ALA was found to inhibit LPS-induced alveolar macrophage death (**Figures 1H, I**). Based on the data presented above, we believe that ALA may reduce the extent of lung injury by inhibiting NETs formation and cell pyroptosis in lung tissue.

### 3.2 NETs induced pyroptosis of macrophages *in vitro*

**Figure 2:**
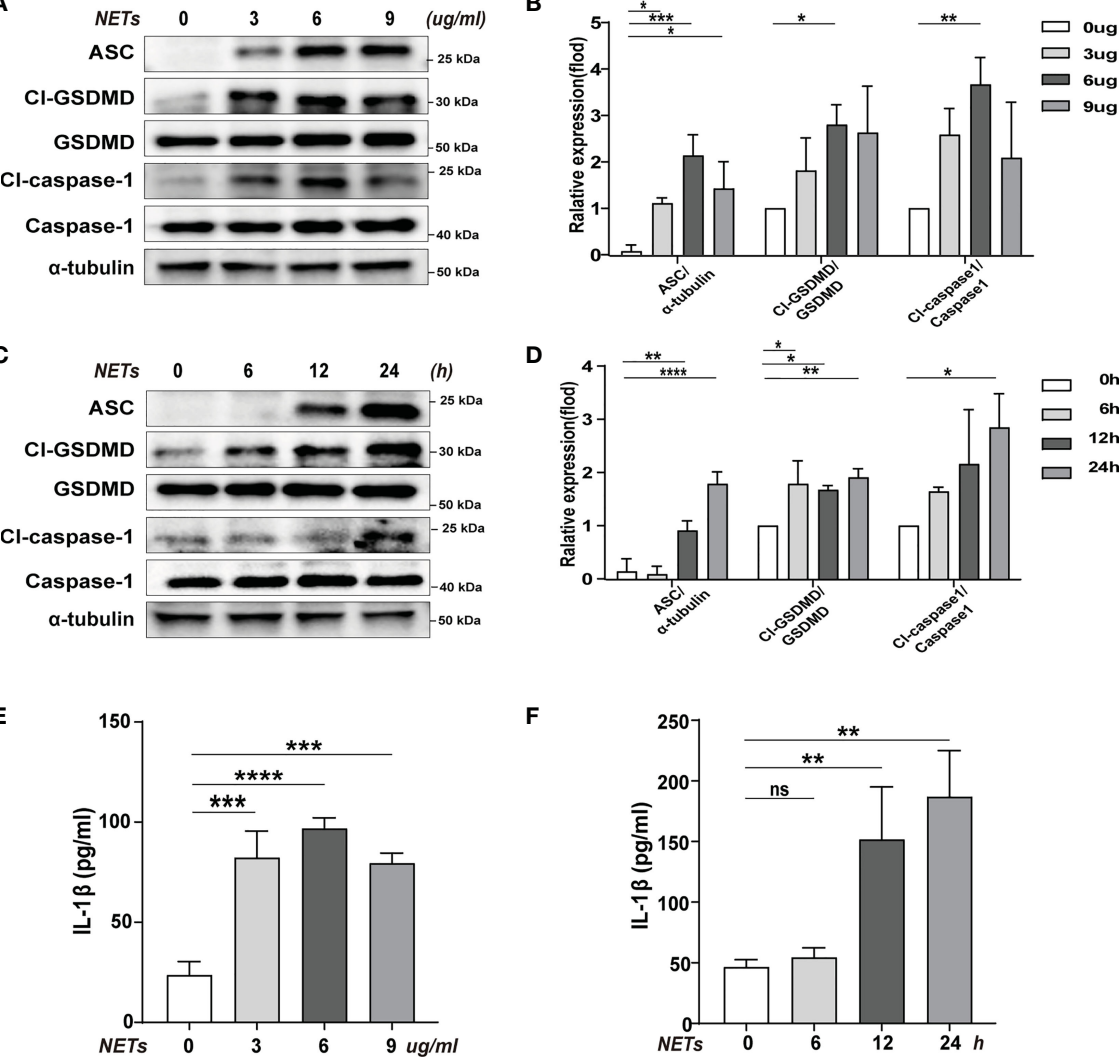
Pyroptosis of macrophages induced by purification of NETs *in vitro*. Bone marrow macrophages(BMDMs)were co-incubated with NETs in concentration gradients (0, 3, 6, 9 ug/ml, 24 h) or time gradients (0, 6, 12, 24 h, 6 ug/ml). **(A–D)** Immunoblot analysis of pyroptosis-related proteins (Cl-caspase-1, Cl-GSDMD, ASC) expression. **(E, F)** Level of inflammatory cytokine IL-1β in cell supernatant was detected by ELISA. Data were represented as mean ± S.D. (n = 3). “ns” represents no significant difference, *P < 0.05, **P < 0.01, ***P<0.001, ****P < 0.0001.

We used purified NETs *in vitro* to incubate with BMDMs to determine the effect of NETs on pyroptosis. ​Using different concentrations of NETs to stimulate the cells, we found that the levels of pyroptosis-related proteins increased to varying degrees when using 3, 6, and 9 ug/ml of NETs, but the most significant increase was seen when the concentration was 6 ug/ml (**Figures 2A, B**). In the meantime, the inflammatory cytokine IL-1β level change was also consistent with the shift in pyroptosis-related proteins (Cl-caspase-1, Cl-GSDMD, ASC) (**Figure 2E**). ​In addition, we have verified temporal gradients of macrophage pyroptosis induced by NETs. ​It was found that the pyroptosis levels of macrophages stimulated by NETs at the same concentration showed a time-dependent variation (**Figures 2C, D**). Similarly, the changing trend of inflammatory cytokine IL-1β was consistent with that of pyroptosis-related proteins (**Figure 2F**). The above data indicated that NETs *in vitro* could induce pyroptosis of macrophages.

### 3.3 ALA inhibited NETs-induced macrophage pyroptosis *in vitro*

**Figure 3:**
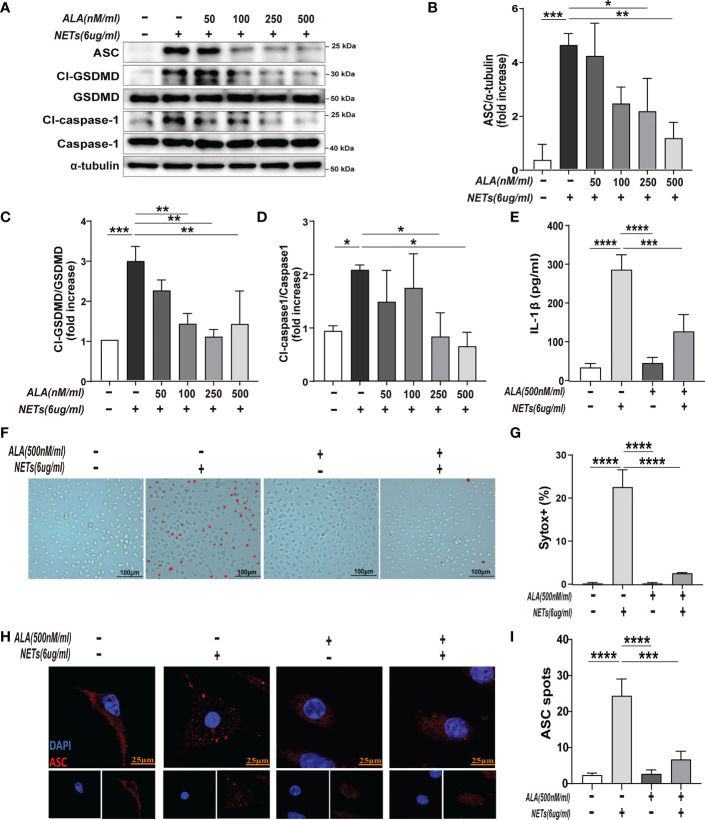
ALA inhibited NETs-induced pyroptosis of macrophages *in vitro*. The bone marrow-derived macrophages (BMDMs) were pretreated with different concentrations of ALA (0, 50, 100, 250, 500 nM/ml), then incubated with NETs for 24 h. **(A–D)** Immunoblot analysis of pyroptosis-related proteins (Cl-caspase-1, Cl-GSDMD, ASC) expression in BMDMs; **(E)** Level of IL-1β in cell supernatant was determined by ELISA; **(F, G)** Immunofluorescence assay for cell death in BMDMs after treatment with NETs (6 μg/ml, 24h); **(H, I)** Images are representative of three independent experiments. Data were represented as mean ± S.D. (n = 3). *P < 0.05, **P < 0.01, ***P<0.001, **** P < 0.0001.

To determine the effect of ALA on macrophage pyroptosis, we pretreated the macrophages with ALA for 2 h and then incubated them with purified NETs for 24 h *in vitro*. We added drug treatment groups with different concentration gradients (0, 50, 100, 250, and 500 nM/ml) to determine whether ALA can inhibit NETs-induced macrophage pyroptosis and to determine the optimal dose of ALA. ​The results showed that NETs enhanced pyroptosis-related proteins (Cl-caspase-1, Cl-GSDMD, ASC) expression, which was inhibited by ALA at concentrations of 250 nM/ml and 500 nM/ml (**Figures 3A–D**). We then tested the role of ALA in inhibiting IL -1β release, macrophage death, and ASC foci formation at a concentration of 500 nM/ml. NETs induced IL-1β release significantly, which was inhibited by ALA (**Figure 3E**). Immunofluorescence staining of macrophage showed the presence of cells positive for Sytox. A large number of Sytox^+^ cells appeared after NETs treatment and were rarely observed with ALA pretreatment (**Figures 3F, G**). Since the ASC protein is a vital junction protein of inflammasome and can aggregate to form the caspase-1 activation platform-the ASC inflammasome (14), immunofluorescence staining was used to observe the formation of ASC inflammasome. It was found that the appearance of ASC inflammasome was evident in the group treated with NETs alone. However, the formation of ASC inflammasome was inhibited by ALA pretreatment (**Figures 3H, I**). Therefore, our results showed that ALA could inhibit NETs-induced macrophage pyroptosis.

### 3.4 Pyrin inflammasome drove NETs-induced macrophage pyroptosis

**Figure 4:**
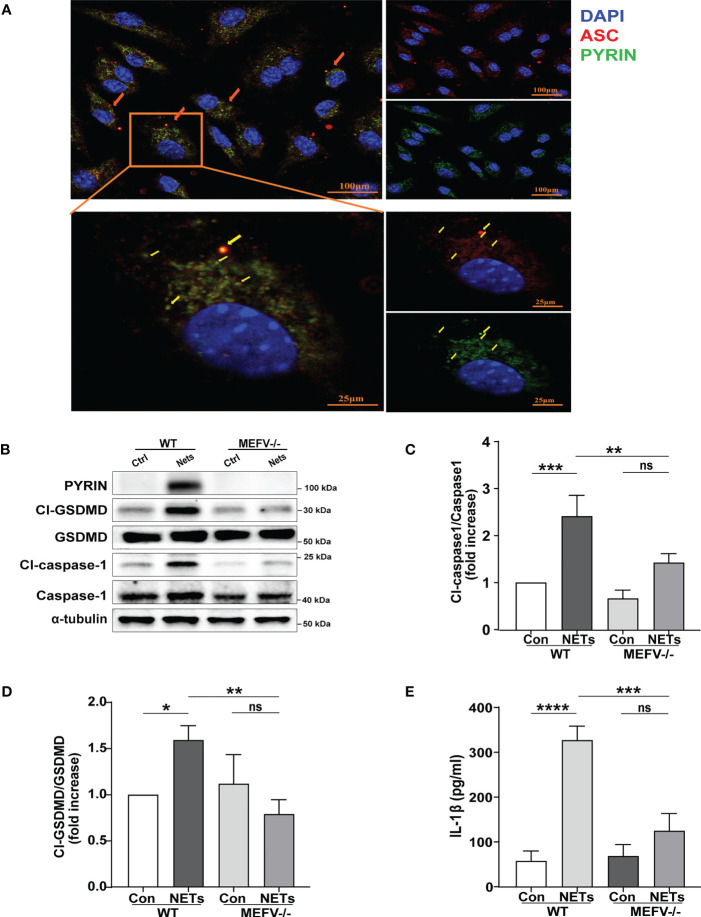
NETs induce macrophage pyroptosis by activating the Pyrin inflammasome. **(A)** Immunofluorescence images of bone marrow-derived macrophages (BMDMs) after treatment with NETs (6 μg/ml, 24h). Arrowheads indicate the ASC/Pyrin speck. Images are representative of three independent experiments. **(B–D)** Immunoblot analysis of pyroptosis-related proteins (Cl-caspase-1, Cl-GSDMD, ASC) expression in the BMDMs from wild-type or Mefv (Pyrin) ^-/-^ mice after treated with NETs (6 μg/ml, 24 h); **(E)** Level of inflammatory cytokine IL-1β in cell supernatant was detected by ELISA. Data were represented as mean ± S.D. (n = 3). “ns” represents no significant difference, *P < 0.05, **P < 0.01, ***P<0.001, ****P < 0.0001.

​ In this study, immunofluorescence staining was used to explore which type of inflammasome drives NETs-induced macrophage pyroptosis. We observed that after NETs stimulation, ​the ASC and Pyrin proteins are clustered into a single bright spot (**Figure 4A**). Therefore, we further selected Mefv (Pyrin)^-/-^ mice to verify the role of Pyrin in driving macrophage pyroptosis. The results showed that in BMDMs from Mefv (Pyrin)^-/-^ mice, the expression of cleaved-caspase-1 and cleaved-GSDMD stimulated by NETs was significantly inhibited compared with the WT group (**Figures 4B–D**), and the level of IL-1β in the cell supernatant was also reduced significantly (**Figure 4E**). All these results indicate that NETs-induced macrophage pyroptosis depended on the Pyrin inflammasome.

### 3.5 Pyrin inflammasome-driven macrophage pyroptosis contributed to sepsis-induced ALI/ARDS

**Figure 5:**
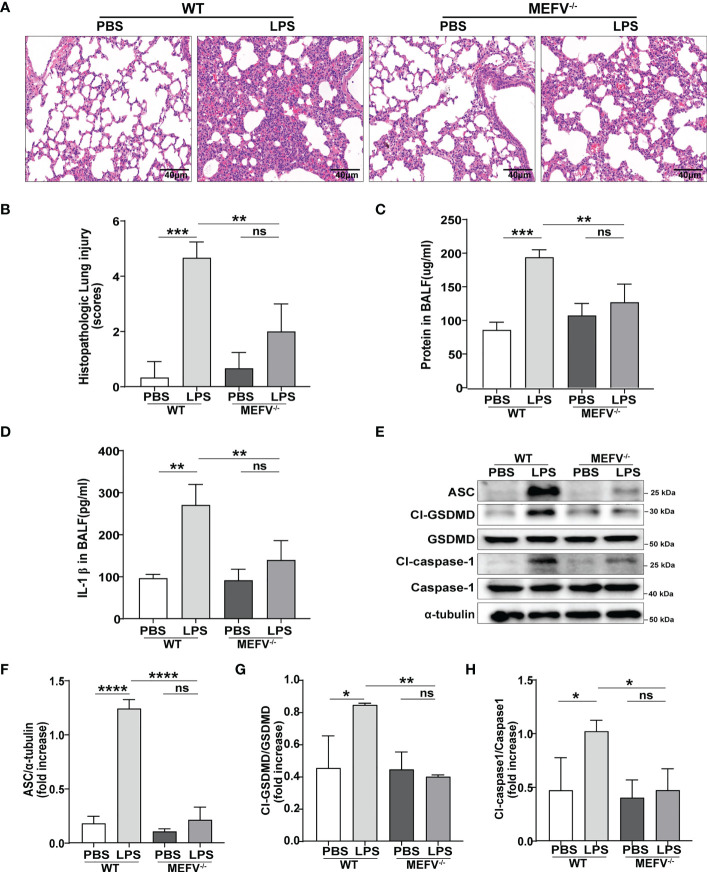
Pyrin inflammasome-driven macrophage pyroptosis contributed to sepsis-induced ALI/ARDS. WT or Mefv (Pyrin) ^-/-^ mice were subjected to ALI/ARDS by intratracheal instillation of LPS (10 mg/kg). At 24 h after LPS instillation, **(A)** haematoxylin and eosin (HE) staining lung tissue sections were examined histologically; **(B)** lung injury pathology score was assessed; **(C)** total protein concentration in BALF was determined by a BCA protein assay kit; **(D)** level of inflammatory cytokine IL-1β in BALF was detected by ELISA; **(E-H)** expression of pyroptosis-related proteins (Cl-caspase-1, Cl-GSDMD, ASC) in lung tissue was analyzed by western blotting. Data were represented as mean ± S.D. (n = 3). “ns” represents no significant difference, *P < 0.05, **P < 0.01, ***P<0.001, **** P < 0.0001.

Mefv (Pyrin)^-/-^ mice were used to investigate the role of Pyrin inflammasome in sepsis-induced ALI/ARDS. Compared to WT mice, LPS induced more slight lung in Mefv (Pyrin)^-/-^ mice examined histologically by HE staining (**Figures 5A, B**). The concentration of protein (**Figure 5C**) and the level of IL-1β (**Figure 5D**) in BALF showed the same results. The expression of pyroptosis-related proteins (Cl-caspase-1, Cl-GSDMD, ASC) in lung tissue examined by western blot was abolished in Mefv (Pyrin)^-/-^ mice (**Figures 5E–H**). All the above data indicated that Pyrin inflammasome-driven macrophage pyroptosis contributed to sepsis-induced ALI/ARDS.

### 3.6 ​ALA inhibited pyrin inflammasome

**Figure 6:**
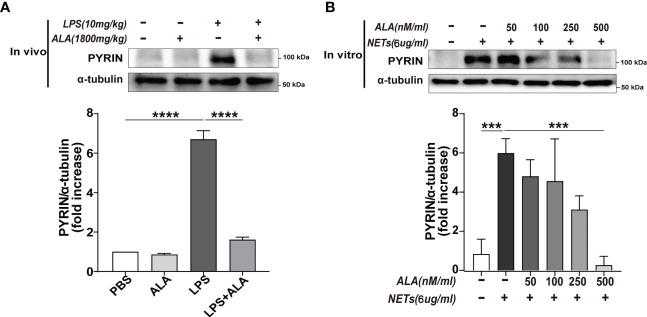
ALA inhibited NETs-induced expression of Pyrin. **(A)** Mice were subjected to ALI/ARDS by intratracheal instillation of LPS (10 mg/kg) and injected intraperitoneally with ALA (1800 mg/kg) or PBS 1 h before LPS instillation. Immunoblot analysis of Pyrin expression in lung tissue at 24 h after LPS instillation. **(B)** Bone marrow-derived macrophages (BMDMs) were pretreated with different concentrations of ALA (0, 50, 100, 250, 500 nM/ml), then incubated with NETs (6 μg/ml) for 24 h. Immunoblot analysis of Pyrin expression in BMDMs. Data were represented as mean ± S.D. (n = 3). ***P<0.001, ****P < 0.0001.

After verifying that Pyrin inflammasome might drive NETs-induced macrophage pyroptosis, we further explored whether ALA could inhibit Pyrin inflammasome-driven pyroptosis. Expression of pyrin was significantly enhanced in lung tissue after LPS treatment, which was inhibited by ALA pretreatment (**Figure 6A**). We also verified that ALA inhibited pyrin expression in a dose-dependent manner *in vitro* (**Figure 6B**). ​Our results showed that ALA could suppress LPS-enhanced expression of Pyrin.

## 4 Discussion

Neutrophils recruited in lung tissue during sepsis can continue to release NETs as described here and in previous work (10). And the excessive release of NETs in lung tissue has been reported to induce macrophage pyroptosis (12, 13). This inflammatory cell death will further amplify the inflammatory response, thus making it difficult to control the disease process (28, 29). In this study, we demonstrate a previously unidentified role of Pyrin inflammasome in driving NETs-induced macrophage pyroptosis. Furthermore, ALA was found to alleviate lung injury by inhibiting Pyrin inflammasome-driven macrophage pyroptosis.

Previous studies pointed out that NETs are essential in mediating lung injury (10, 30, 31). In clinical studies, the level of NETs in BALF of patients with ARDS was significantly increased, and the increased level was positively correlated with the severity of the disease (10). In addition, animal experiments also found that, in the sepsis model, the level of NETs in the BALF of mice also increased significantly (32), and the increased level of NETs was related to the proportion of pyroptosis of alveolar macrophages (13). Using DNase I(An endonuclease that digests DNA) to inhibit the generation of NETs could significantly reduce cell pyroptosis, thereby reducing the inflammatory response and improving the survival rate (28). In this study, we also found that the level of alveolar NETs was significantly increased in the sepsis mice model, and the extent of pyroptosis in lung tissue was increased significantly. This phenomenon has also been confirmed *in vitro*. We found that NETs can induce BMDMs pyroptosis in a concentration and time-dependent manner. Consistent with other studies (12, 13), these results indicated that NETs-induced alveolar macrophage pyroptosis played an essential role in ARDS. ​Therefore, it is significant to find a method to inhibit alveolar macrophage pyroptosis in ALI/ARDS.

Our previous study reported the lung protection effect of ALA, mainly focusing on inhibiting the expression of inflammatory factors, including pyroptosis-related IL-1β (23). ​ However, the underlining mechanism by which ALA suppresses the expression of inflammatory factors remains unknown. Known to all, IL-1β is a major cytokine involved in macrophage pyroptosis. Our previous study also found that macrophage pyroptosis contributed to the excessive inflammation and cytokine storm in ARDS (8). Therefore, whether ALA can inhibit macrophage pyroptosis and ARDS was investigated in this study. ​This is the first time that ALA has been found to inhibit macrophage pyroptosis in ARDS achieving lung protection.

​ALA has been reported to reduce neutrophil counts in the lungs (33). Meanwhile, we also found that ALA can reduce the level of NETs in BALF. ​However, the level of NETs in the ALA+LPS group decreased by only 28% compared to the LPS group. Therefore, ​the effect of ALA in reducing the NETs level in BALF was minor. We then explored whether ALA could directly inhibit NETs-induced pyroptosis *in vitro*. The results showed that ALA could inhibit NETs-induced pyroptosis in a concentration-dependent manner. Moreover, ALA was able to impede pyroptosis at a higher concentration completely. The above results indicated that ALA not only inhibited the generation of NETs but also directly inhibited NETs-induced pyroptosis, which contributed to the main effect of ALA.

Next, our research focuses on how ALA inhibits NETs-mediated pyroptosis. ​It is known that pyroptosis, a form of programmed cell death, relies on the assembly of the inflammasome to activate downstream caspases and GSDMD (16). A wide range of currently studied inflammasomes includes the family of nucleotide-binding domain leucine-rich repeat (NLR) proteins (NLRP1, NLRP3, NLRP7, and NLRC4), the protein absent in melanoma 2 (AIM2), and Pyrin (14). ​ Pyrin may function as an innate immune ‘guard’ in antimicrobial defense, which has been poorly investigated in the sepsis-induced ALI/ARDS. While the activation of other inflammasomes is inhibited, it conversely potentiates pyrin inflammasome activation (17). In our study, we were surprised to find that when NETs act on macrophages to induce pyroptosis, the Pyrin inflammasome is activated. We found that pyroptosis was completely inhibited in NETs-stimulated BMDMs from Mefv (Pyrin)^-/-^ mice. The same results were found in the LPS-challenged lung tissue from Mefv (Pyrin)^-/-^ mice. In addition, LPS-induced lung injury was found slight in the lung tissue from Mefv (Pyrin)^-/-^ mice. These results indicated that NETs-induced macrophage pyroptosis depended on the activation of Pyrin inflammasome, which contribute to sepsis-induced ALI/ARDS. After confirming that Pyrin inflammasome plays a role in NETs-induced pyroptosis, we further found that the expression of Pyrin protein decreased significantly with ALA pretreatment, which was confirmed *in vivo* and *in vitro*. These results imply that ALA might inhibit macrophage pyroptosis by inhibiting Pyrin expression.

NETs is a complex reticular structure composed of histones, DNA, and various proteases: myeloperoxidase (MPO), neutrophil elastase (N.E.), etc. (34). So, its pathway to induce pyroptosis of macrophages may be very complex. We also investigated the role of classical NLRP3 inflammasome in NETs-induced macrophage pyroptosis. ​Although the expression of NLRP3 protein was higher in LPS-challenged lung and NETs-challenged macrophage, both were recovered by ALA (**Supplementary Figures 1A–D**). However, ​Knockout of the NLRP3 gene did not affect NETs-induced macrophage pyroptosis (**​​Supplementary Figure 1E**), suggesting that NLRP3 inflammasome is independent of NETs-induced macrophage pyroptosis. ​The role of ALA in inhibiting macrophage pyroptosis was also not linked to NLRP3 inflammasome.

In addition, AIM2 is a direct receptor for cytosolic double-strand DNA (35). Silencing of the AIM2 gene was reported to protect against NETs-induced macrophage pyroptosis (13). This study found that ALA did not mediate AIM2 expression *in vivo* and in Vitro (​**Supplementary Figure 2**). Based on the above results, we conclude that ALA alleviates ALI/ARDS mainly through inhibiting Pyrin-driven macrophage pyroptosis. Further studies are needed to explore the role of other inflammasomes in driving NETs-induced macrophage pyroptosis and whether there is a synergistic effect between different inflammasomes.

Our work is focused on whether pretreatment of ALA could inhibit NETs-induced macrophage pyroptosis in sepsis-induced ALI/ARDS, as well as to identify which inflammasome is involved in this process. ​However, the therapeutic potential of ALA will be equally important for people already suffering from sepsis-induced ALI/ARDS. Therefore, we will investigate the therapeutic potential of ALA in mice after tracheal administration of LPS in future studies.

## 5 Conclusions

**Figure 7:**
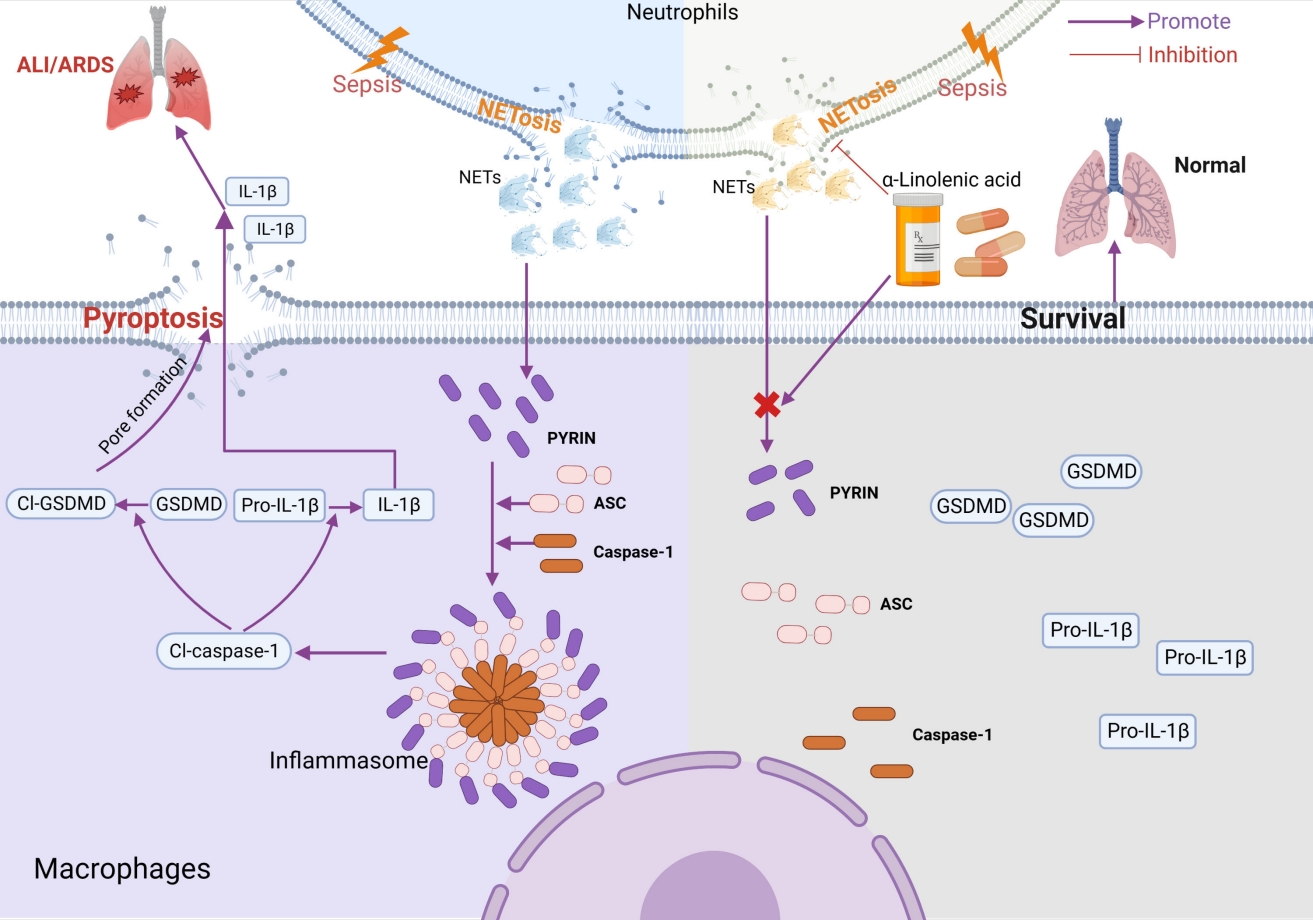
Model for ALA alleviating macrophage pyroptosis in sepsis-induce ARDS. In sepsis-induced ALI/ARDS, Pyrin inflammasome drove NETs-induced alveolar macrophage pyroptosis. ALA might alleviate ALI/ARDS by inhibiting Pyrin inflammasome activation. Created with BioRender.com.

Based on this study, we propose a mechanistic model for ALA alleviating macrophage pyroptosis in ARDS (**Figure 7**). Pretreatment of ALA might alleviate NETs-induced alveolar macrophage pyroptosis by mediating Pyrin inflammasome activation, which contributes to the mechanism by which ALA relieves ALI/ARDS. This study provides a new therapeutic target for treating ALI/ARDS, for which ALA may be a potential therapeutic.

## Data availability statement

The original contributions presented in the study are included in the article/**Supplementary Material**. Further inquiries can be directed to the corresponding authors.

## Ethics statement

The animal study was reviewed and approved by The Ethics Committee of Shanghai General Hospital, Shanghai Jiaotong University School of Medicine (No. 2019AW009).

## Author contributions

ZY and JL designed the study. CL drafted the manuscript. CL, YZ, QT, LY contributed to data acquisition and analysis. All authors contributed to the article and approved the submitted version. 

## Supplementary material

Supplementary Figure 1 The role of NLRP3 in the process of ALA alleviating ALI/ARDS. **(A, B)** Mice were subjected to ALI/ARDS by intratracheal instillation of LPS (10 mg/kg) and injected intraperitoneally with ALA (1800 mg/kg) or PBS 1 h before LPS instillation. Immunoblot analysis of NLRP3 expression in lung tissue at 24 h after LPS instillation. **(C, D)** Bone marrow-derived macrophages (BMDMs) were pretreated with different concentrations of ALA (0, 50, 100, 250, 500 nM/ml), then incubated with NETs (6μg/ml) for 24 h. Immunoblot analysis of NLRP3 expression in BMDMs. **(E)** BMDMs from wide type (WT) or NLRP3^-/-^ mice were incubated with NETs (6μg/ml) for 24 h. Immunoblot analysis of NLRP3 expression in BMDMs.Data were represented as mean ± S.D. (n = 3). **P<0.01, ***P<0.001, **** P < 0.0001.

Supplementary Figure 2 The role of AIM2 in the process of ALA alleviating ALI/ARDS. **(A, B)** Mice were subjected to ALI/ARDS by intratracheal instillation of LPS (10 mg/kg) and injected intraperitoneally with ALA (1800 mg/kg) or PBS 1 h before LPS instillation. Immunoblot analysis of AIM2 expression in lung tissue at 24 h after LPS instillation. **(C, D)** Bone marrow-derived macrophages (BMDMs) were pretreated with different concentrations of ALA (0, 50, 100, 250, 500 nM/ml), then incubated with NETs (6μg/ml) for 24 h. Immunoblot analysis of AIM2 expression in BMDMs. Data were represented as mean ± S.D. (n = 3). *P<0.05.

Supplementary Figure 3 Representative flow cytometry gating strategy to assess macrophages. Gate for nucleated cells based on the size and complexity of the event (FCS-A and SSC-A, respectively). Nucleated cells were further plotted in FSC-A and FSC-H to gate single cells and exclude doublets. From the single cell gate, macrophages were defined as F4/80^+^.
