# Supplementary figures and images for: Alpha-linolenic acid pretreatment alleviates NETs-induced alveolar macrophage pyroptosis by inhibiting pyrin inflammasome activation in a mouse model of sepsis-induced ALI/ARDS
Source: Front Immunol. 2023 Mar 27;14:1146612. doi: 10.3389/fimmu.2023.1146612 (PMC10083395; doi:10.3389/fimmu.2023.1146612)

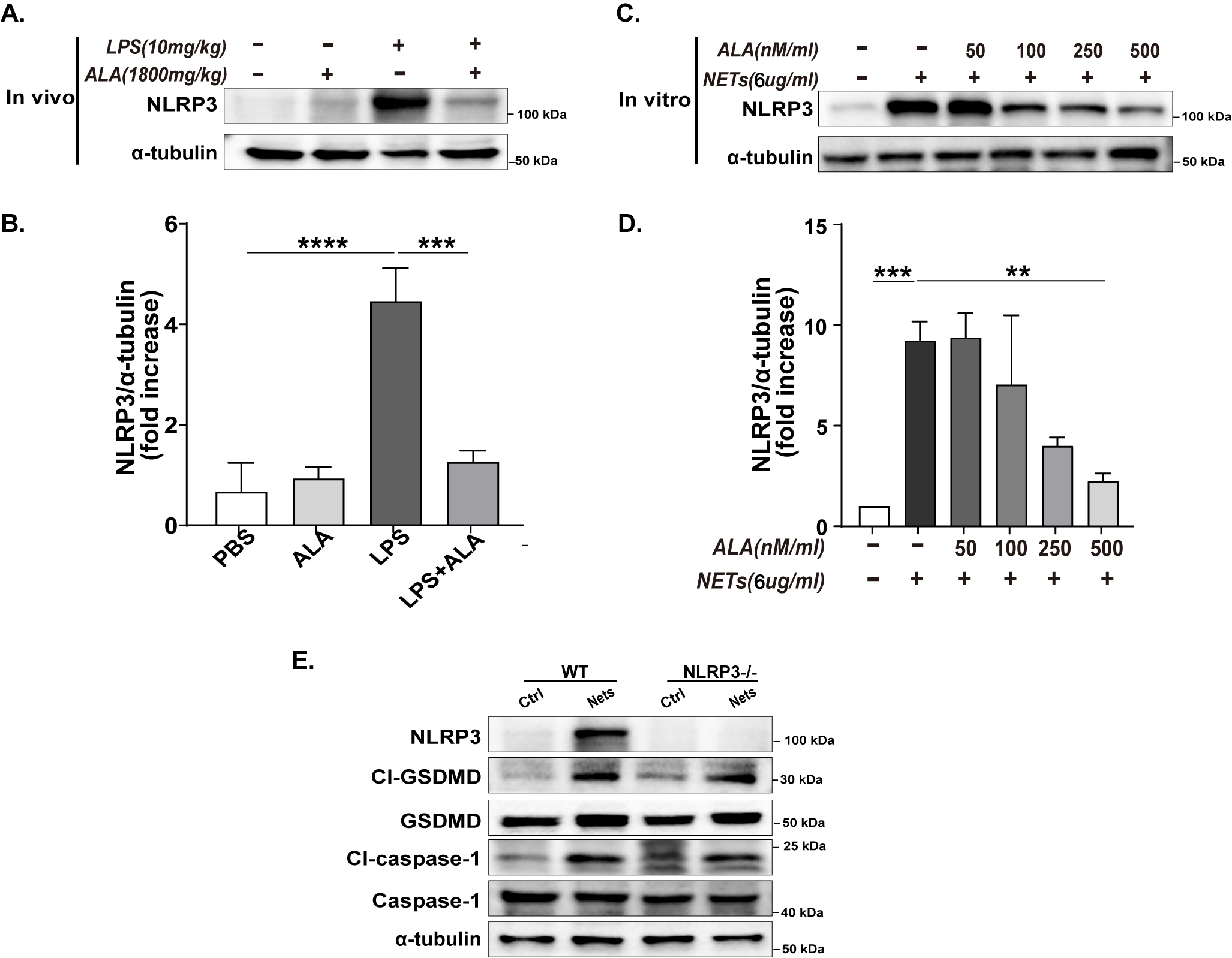

Supplement: Supplementary Figure 1 — The role of NLRP3 in the process of ALA alleviating ALI/ARDS. (A, B) Mice were subjected to ALI/ARDS by intratracheal instillation of LPS (10 mg/kg) and injected intraperitoneally with ALA (1800 mg/kg) or PBS 1 h before LPS instillation. Immunoblot analysis of NLRP3 expression in lung tissue at 24 h after LPS instillation. (C, D) Bone marrow-derived macrophages (BMDMs) were pretreated with different concentrations of ALA (0, 50, 100, 250, 500 nM/ml), then incubated with NETs (6μg/ml) for 24 h. Immunoblot analysis of NLRP3 expression in BMDMs. (E) BMDMs from wide type (WT) or NLRP3-/- mice were incubated with NETs (6μg/ml) for 24 h. Immunoblot analysis of NLRP3 expression in BMDMs.Data were represented as mean ± S.D. (n = 3). **P<0.01, ***P<0.001, **** P < 0.0001. [file Image_1.tif]

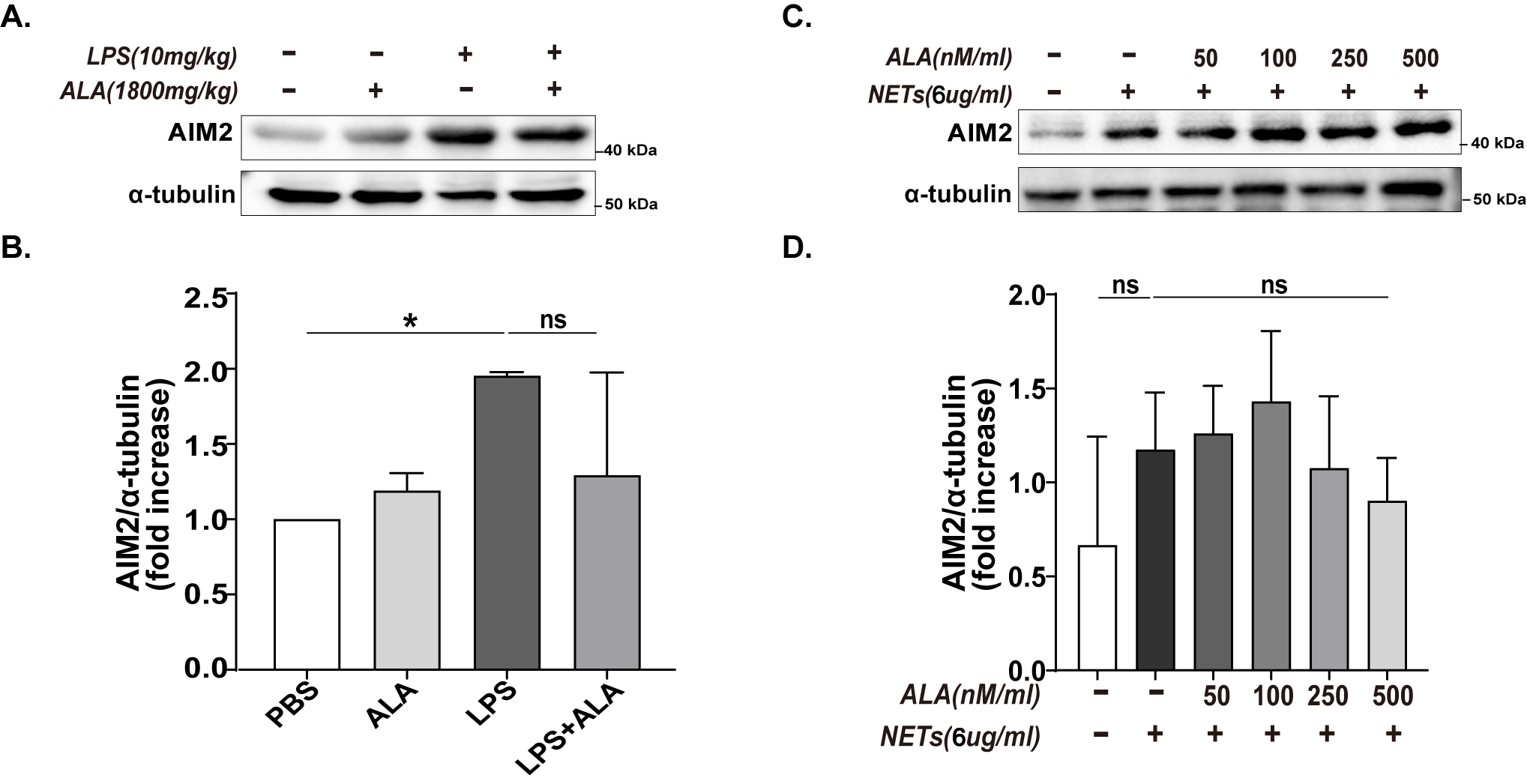

Supplement: Supplementary Figure 2 — The role of AIM2 in the process of ALA alleviating ALI/ARDS. (A, B) Mice were subjected to ALI/ARDS by intratracheal instillation of LPS (10 mg/kg) and injected intraperitoneally with ALA (1800 mg/kg) or PBS 1 h before LPS instillation. Immunoblot analysis of AIM2 expression in lung tissue at 24 h after LPS instillation. (C, D) Bone marrow-derived macrophages (BMDMs) were pretreated with different concentrations of ALA (0, 50, 100, 250, 500 nM/ml), then incubated with NETs (6μg/ml) for 24 h. Immunoblot analysis of AIM2 expression in BMDMs. Data were represented as mean ± S.D. (n = 3). *P<0.05. [file Image_2.tif]

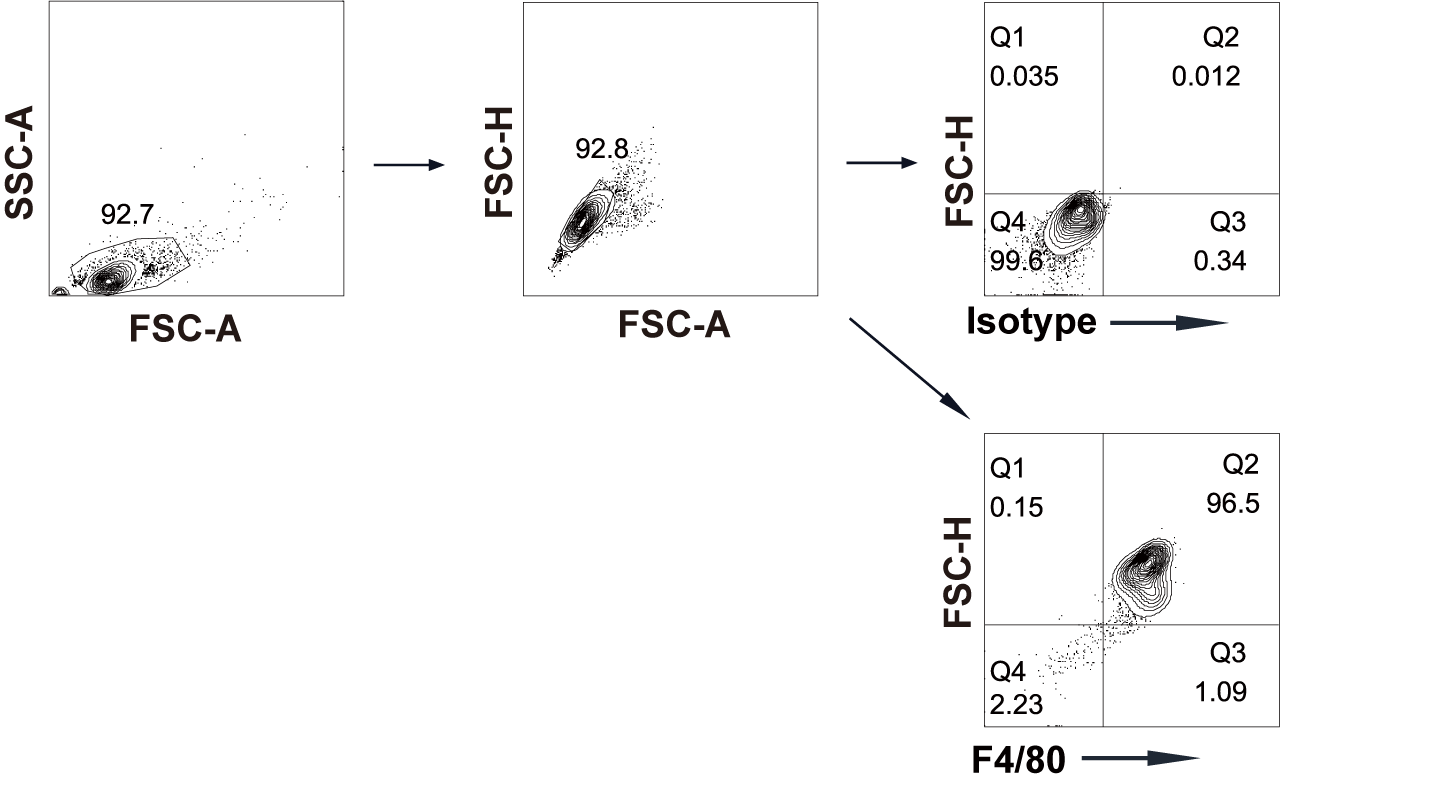

Supplement: Supplementary Figure 3 — Representative flow cytometry gating strategy to assess macrophages. Gate for nucleated cells based on the size and complexity of the event (FCS-A and SSC-A, respectively). Nucleated cells were further plotted in FSC-A and FSC-H to gate single cells and exclude doublets. From the single cell gate, macrophages were defined as F4/80+. [file Image_3.tif]
